# The relationship between alexithymia, empathy, willingness to fulfill the contract, and communication skills attitudes of rural-oriented tuition-waived medical students of China: a cross-sectional study

**DOI:** 10.3389/fmed.2025.1648789

**Published:** 2025-09-26

**Authors:** Lining Zhang, Yuxuan Xu, Leyan Gao, Chen Feng, Guanqi Han, Xuewen Zhang, Xintong Xu, Aimin Tang

**Affiliations:** ^1^Faculty of Education, Qufu Normal University, Qufu, China; ^2^Jining Medical University, Jining, China; ^3^College of Clinical Medicine, Jining Medical University, Jining, China

**Keywords:** RTMSs, communication skills attitude, empathy, willingness to fulfill the contract, alexithymia

## Abstract

**Background:**

Rural-oriented tuition-waived medical students (RTMSs) with good communication abilities can help avoid medical conflicts and ensure high-quality rural medical services. This study evaluated the influence of empathy, willingness to fulfill the contract, and alexithymia on RTMSs’ communication skills attitude, and explored the mediating roles of empathy.

**Methods:**

This cross-sectional study, conducted between 3 and 27 April 2024, randomly selected three medical universities in Shandong Province responsible for training RTMSs, with 495 students enrolled as participants. The Jefferson Scale of Empathy-Student (JSPE-S), Communication Skills Attitude Scale (CSAS), Willingness to Fulfill the Contract Scale, and the 20-item TAS-20 were used for investigation. Statistical methods such as one-way ANOVA, exploratory factor analysis (EFA), bivariate correlation, and structural equation modeling (SEM) were used.

**Results:**

Alexithymia had a significant direct negative impact on empathy (*β* = −0.26, *p* < 0.001). It also negatively affected communication skills attitude (*β* = −0.17, *p* < 0.001). Additionally, it reduced the willingness to fulfill the contract (*β* = −0.26, *p* < 0.001). Willingness to fulfill the contract had a significant direct positive impact on empathy (*β* = 0.26, *p* < 0.001) and communication skills attitude (*β* = 0.09, *p* < 0.001). In the mediation pathway between willingness to fulfill the contract and communication skills attitude, empathy had a significant mediating effect, 95% CI: 0.6602–1.7526. In the mediating pathway between alexithymia and communication skills attitude, empathy also had a significant mediating effect [95% CI: (−0.8955) − (−0.3292)].

**Conclusion:**

Schools and policy makers can strengthen RTMSs’ professional ethics, career identity, and professional identity education, increase RTME policy publicity and public opinion guidance, strengthen RTME policy support and restraint measures, help RTME strengthen the willingness to fulfill the contract, overcome alexithymia, play the role of empathy as an intermediary, and then improve the communication skills attitude of RTMSs. This survey can provide a feasible new plan for promoting the construction of a good doctor–patient relationship and the high-quality development of rural medical service.

## Introduction

The uneven development of medical and health services in urban and rural areas, unreasonable resource allocation, and the comparatively weak medical and health work in rural areas have always been the most prominent problems plaguing China’s healthcare service system (1). As urbanization accelerates, the gap in medical resources between urban and rural areas is widening. Rural regions face shortages of medical resources and low service levels, which struggle to meet basic healthcare needs, severely impacting rural residents’ health and quality of life. Meanwhile, China’s rapid economic growth has led to rapid population aging, with rural elderly people receiving significantly less medical care and services than their urban counterparts. Statistics show that the prevalence of chronic diseases among rural elderly people is 68.93%, and the rates of BADL and IADL impairment are 14.40 and 47.16%, respectively (2), all much higher than those of urban elderly people. This highlights a more severe aging situation in rural areas.

To address these issues, in 2010, China’s National Development and Reform Commission and other departments jointly issued a notice to implement free training for rural-oriented tuition-waived medical students (RTMSs), officially launching the rural-oriented tuition-waived medical education (RTME) project. The goal is to train healthcare professionals for general practice in rural township health centers and below. RTMSs must sign targeted employment agreements with universities and local health administrations and serve in primary health care institutions for 6 years after graduation. This policy effectively addresses the talent shortage in the primary healthcare team (3). From 2015 to 2023, about 50,000 rural-bound medical students who graduated according to the contract have provided medical and health services in rural areas (4).

Despite its well-designed nature, the implementation of RTME program is unsatisfactory. Issues such as backward rural primary medical institutions, limited career development, and low social recognition have led many primary medical service providers to waver in their commitment to targeted employment. For example, among the 950 RTMSs trained by Chongqing Medical University since 2010, 302 (31.8%) have a serious tendency to default and do not want to work in contracted rural areas, due to reasons including long service hours (27.60%, 85 out of 308) and low pay (14.94%, 46 out of 308) (5). Our research group investigated 1,162 RTMSs from four medical colleges in Shandong Province in 2023 and found that 26.4% of RTMSs had low willingness to fulfill contracts (6). The wavering of the performance of the contract may lead to poor service quality, humanistic care, and communication ability in future rural medical institutions after RTMSs’ graduation, which will further affect the implementation and sustainable development of RTME.

In 2001, the International Medical Education Professional Committee issued the Global Minimum Basic Requirements for Medical Education, emphasizing “communication skills” as one of the seven macro-level teaching achievements and abilities (7). Under the modern medical model, the lack of doctors’ communication ability not only directly affects the quality of communication between doctors and patients, but also indirectly affects the communication and cooperation within the medical team (7). In 2002, the International Standards for Medical Education, formulated by the International Association for Global Medical Education, and the Basic Requirements for Undergraduate Teaching of Clinical Medicine, formulated by China in 2003, both emphasized the communication and exchange ability of medical students and formulated guiding and operational standards for this field (8). Medical schools all over the world take communication skills training as an important part of medical education. For example, in the United States, communication skills are considered to be one of the basic skills that medical students should possess. The British Medical Association (BMA) assesses doctors’ communication skills as part of their qualification exams; The National Committee of Medical Education of China regards the effective communication ability of medical students as one of the important norms of medical education (9). Since 2019, Guangxi Medical University has offered a stepped general practice communication skills course from simple to difficult to cultivate the doctor–patient communication skills of RTMSs (10).

In summary, effective communication skills are crucial for medical students’ future practice, especially for rural town medical students (RTMSs) who will serve rural residents. These residents often lack health education and have a limited understanding of medical terminology, while trust in doctors significantly influences their healthcare decisions. Simplifying medical explanations can enhance doctor–patient communication, build a professional and reliable physician image, and increase patient trust and acceptance of medical advice and treatment plans. This, in turn, boosts doctors’ professional satisfaction and commitment to rural healthcare, supporting the sustainability of the rural town medical education (RTME) project.

Given that attitudes toward communication skills directly impact training effectiveness, fostering a positive attitude is essential for improving communication skills. In order to determine whether medical students need communication skills training, it is necessary to evaluate the communication skills and attitude of medical students (11). Professor Rhys C. from the University of Dundee, UK, developed and validated the Communication Skills Attitude Scale (CSAS) (11–13). Effective doctor–patient communication skills, rooted in a positive attitude, play a vital role in mitigating medical disputes, enhancing patient compliance, and ensuring medical efficiency. When communicating with patients, if the doctor’s communication ability is poor, it will lead to doctor–patient communication barriers or even wrong communication, so that the doctor lacks self-confidence, and even questions the treatment plan, which will affect their positive attitude to communicate with patients over time. It is found that the communication skills and attitude of the RTMSs will be affected by various factors, such as the willingness to fulfill the contract, alexithymia, and empathy (14).

Empathy enables observers to understand others’ thoughts and situations, positively impacting doctor–patient communication and trust-building. Medical personnel with high empathy can better perceive patients’ psychological and emotional changes, understand their behaviors, and adjust their communication methods. Additionally, empathy from doctors makes patients feel accepted, understood, and respected, reducing anxiety and enhancing the trust between doctors and patients. This improves treatment effectiveness and doctors’ sense of accomplishment (15). Empathy is the foundation of the patient-centered approach in the practice of grassroots general medical care. Related studies have shown that most patients will recommend empathetic doctors to other individuals (16). A survey conducted by Tongji University among 1958 clinical medicine students in the Shanghai area proved that cultivating empathy skills can promote the students’ positive attitudes toward communication skills (14). In this study, it is assumed that empathy has a positive impact on the communication skills attitude of RTMSs, and the relationship between them is verified, with the expectation of providing more theoretical support for improving the communication skills attitude from the perspective of empathy skills.

Working conditions, working environment, and industry prospects are factors that affect the willingness of RTMSs to fulfill contracts, and a low willingness to serve in rural areas after graduation can be inferred that medical students will be less motivated to learn medical communication skills at school, making them unable to play a good role as primary doctors. Research has shown that the empathy ability of medical staff is negatively correlated with turnover intention (17), which can be understood as a positive correlation between empathy and willingness to fulfill their contract of RTMSs. Therefore, it is very necessary to further explore the relationship between willingness to fulfill their contract, empathy, and communication skills attitude, providing a reference for improving the quality of RTMSs’ training.

Alexithymia is a phenomenon in which individuals find it difficult to express and recognize emotions. However, much research on RTMSs treats occupational burnout and depressive symptoms as pivotal outcomes; they are, in fact, common sequelae of chronic occupational stress; alexithymia, by contrast, is a trait-level deficit in emotional awareness that both precedes and prospectively predicts these conditions. In addition, compared to clinical depression, the stigmatization of screening for affective disorders is lower, and the response to skill-based brief interventions is more sensitive. Therefore, according to the task of the National Health Commission in 2023, which is to enhance “emotional ability” as a memory strategy, we have identified alexithymia as a key psychological variable in this study. Due to the particularity of the medical profession, alexithymia affects medical students’ empathy ability, communication skills and attitudes, and thus affects their doctor–patient communication ability and clinical diagnosis and treatment ability. Moriguchi et al. (18) found that the more severe the alexithymia of the subjects, the more difficult the perception of pain. Meanwhile, in the study of patients with depression, it was found that the scores of the Interpersonal Relationship Indicator Scale (IRI) and multidimensional empathy paradigm (MET) of patients with high alexithymia were generally lower than those of patients with low alexithymia. That is, the emotional and empathic dimensions of patients with hyper-alexithymia are impaired (19, 20). In addition, the same research on college students has reached the same conclusion (18). Through the understanding of RTMSs’ alexithymia, this study can reveal the characteristics of alexithymia in this population, and provide mental health education for individuals with alexithymia to help them improve their ability of emotion recognition, expression, and regulation. At the same time, by exploring the relationship between alexithymia and attitude toward communication skills, more ways to improve communication skills can be found. Studies have shown that alexithymia is the strongest (negative) predictor of empathy (21). This study also hopes to influence the level of empathy in RTMSs by regulating alexithymia; so, alexithymia is a factor worth focusing on.

Based on the emotional cognitive processing chain theory framework, this study constructed a five-path hypothesis model that includes three direct paths and two mediating paths. Alexithymia, characterized as a “defect in emotional recognition,” impairs an individual’s ability to label and express their own emotions. This defect not only directly suppresses empathy abilities (covering the three core dimensions of opinion-taking factor, compassionate care factor, and transposition factor), but also directly negatively predicts communication skills attitude. The willingness to fulfill the contracts has a significant positive direct impact on both empathy and communication skills attitude. Empathy presents a dual mediating effect in the model: it not only transmits the inhibitory effect of alexithymia on communication skills attitude, but also enhances the promoting effect of willingness to fulfill the contracts on communication skills attitude. This mechanism fully confirms the cascade processing chain of emotion recognition → empathy understanding → behavioral attitude, systematically explains the mechanism by which emotional cognitive deficits gradually weaken the capacity of primary healthcare, and provides theoretical targets for the development of targeted intervention measures.

**Figure 1:**
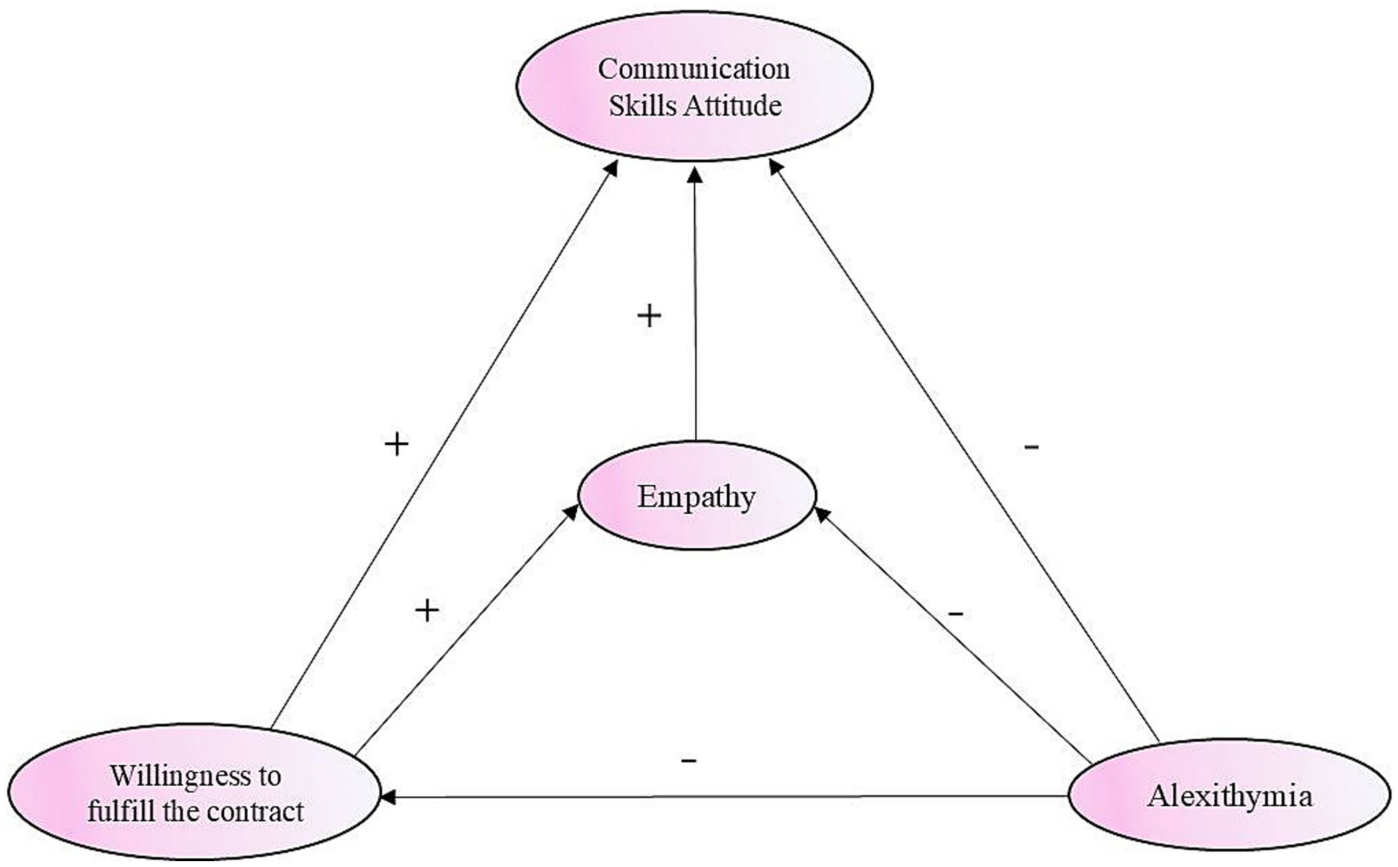
Hypothesized conceptual model of communication skills attitude among empathy, alexithymia, and willingness to fulfill the contract.

To sum up, this SEM-based study addresses two clearly defined research questions: Do RTMSs’ willingness to fulfill the contract and alexithymia directly predict their attitudes toward learning communication skills? Does empathy mediate these relationships? The model, as specified in Table 1 and illustrated in Figure 1, provides valuable insights. The results offer a reference for the cultivation and ability improvement of RTMSs, highlighting the critical role of empathy in mediating the effects of emotional deficits and motivational factors on communication skills attitude.

**Table 1:** Theoretical hypotheses.

Hypotheses
1. RTMSs’ empathy positively impacts communication skills attitude
2. RTMSs’ willingness to fulfill the contract positively impacts their communication skills attitude
3. RTMSs’ alexithymia negatively impacts communication skills attitude
4. RTMSs’ willingness to fulfill the contract indirectly positively impacts communication skills attitude through the mediating effect of empathy
5. RTMSs’ alexithymia indirectly negatively impacts communication skills attitude through the mediating effect of empathy

## Methods

### Participants and procedures

In 2017, China’s Shandong province launched the RTME program, which aims to train general practitioners for rural areas after 5 years of education and welcome the first batch of medical graduates in 2022. This study employed a multi-stage random sampling strategy to obtain the research sample. The specific procedures were as follows: (1) Institutional sampling: Based on simple random sampling principles, three undergraduate medical universities in Shandong Province responsible for training RTMSs (Jining Medical University, Binzhou Medical University, and the Second Medical University of Shandong) were randomly selected as research sites. (2) Stratification and class framework construction: RTMSs from the three universities were stratified by academic year into senior cohort (interns and trainees with clinical experience), intermediate cohort (second- and third-year students who had completed partial medical theory coursework), and junior cohort (first-year students without systematic exposure to medical knowledge). Institutional records indicated these universities contained six senior, six intermediate, and 3 junior administrative classes, respectively. (3) Classroom sampling: Using simple random sampling, three classes were randomly selected from each of the six senior and intermediate cohorts. All three classes in the junior cohort were included without further sampling. (4) Participant identification and screening: Cluster sampling was applied to include all students from the three selected classes per cohort (all three junior classes). This yielded an initial pool of 521 RTMSs. Based on predetermined exclusion criteria: (1) individuals explicitly refusing survey participation; (2) individuals declining to sign the contract agreement; (3) individuals with unregistered or withdrawn student status. Fourteen students were excluded.

The built-in validity indicators of the mature scales used in this study, especially the TAS-20, provided indirect attention detection based on reverse items and item consistency. Meanwhile, this study implemented various methods for quality control, including response pattern (regular response detection, and extreme value checking), response time, proportion of missing values, and logical consistency checks. A total of 507 students entered the survey phase, and 495 valid questionnaires were ultimately collected, with a valid questionnaire response rate of 97.63%.

In this cross-sectional study, all measurement tools (such as the TAS-20, JSPE-S, Willingness to Fulfill the Contract Scale, and CSAC) were distributed at the same time point (specifically organized meeting time), presided over and supervised by the researchers, and filled out and collected on-site through a one-time anonymous self-administered questionnaire for data collection. To ensure data quality, researchers focused on eliminating participants’ concerns and encouraging genuine responses through strict anonymous confidentiality measures. By providing sufficient informed consent, emphasizing importance, arranging processes reasonably, and training the main participants, we aimed to improve participation compliance and honesty; and after data collection, strict screening and cleaning processes (including logical checks, handling missing values, and identifying and removing invalid questionnaires) were carried out to ensure the reliability and validity of the final data used for analysis.

The ethical review was solicited from the Ethics Committee of Jining Medical University by the project team (Approval Number: JNMC-2022-YX-030), and the expert group issued the ethical consent certificate after reviewing the materials. Investigators were required to inform participants of the anonymous nature and the purpose of the study before conducting the survey, and to obtain their verbal consent before conducting data collection. Only after participants had verbally confirmed their consent were they allowed to receive the questionnaire. This consent process was recorded by the principal investigator, and a statement was signed.

### Measures

A questionnaire was designed based on the survey questionnaire of career obstacles among Chinese college students (22), consisting of five parts: the first part included demographic characteristics (gender, age, student origin, etc.) and academic characteristics (learning motivation, academic plan, learning goals, etc.). The second to fifth parts included the Communication Skills Attitude Scale (CSAS), the Toronto Alexithymia Scale (TAS-20), the Willingness to Fulfill the Contract Scale, and the Jefferson Scale of Physician Empathy-Student Version (JSPE-S).

### Communication Skills Attitude Scale

Rees et al. (23) realized that the negative emotions of medical students seriously affected the effectiveness of medical students’ communication skills learning, so they developed the Medical Students’ Communication Skills Attitude Scale (CSAS), which was later translated into Chinese by Tian et al. The scale consists of 26 items, including two dimensions of positive attitude (PAS) and negative attitude (NAS). The Cronbach’s *α* coefficients of the two dimensions are 0.85 and 0.74, respectively (11). PAS and NAS are 5-point Likert scales, with a total score ranging from 13 to 65 points; the higher the score, the stronger the attitude.

### Jefferson Scale of Physician Empathy-Student Version

At present, the Interpersonal Reactivity Index (IRI) developed by Davis is commonly used to measure the empathy of ordinary college students, but its disadvantage is that it is not applicable to doctor–patient situations (24–26). Therefore, the Jefferson Doctor Empathy Scale (JSE), developed by Dr. Mohammadreza Hojat, was revised into Chinese by Jiang Tian and used to measure empathy in RTMS (27). With Cronbach’s *α* coefficient of 0.861, the scale was composed of three dimensions: perspective selection (10 items), sympathy (eight items), and perspective adoption (two items). The answers for each item were set on a 7-point Likert choice scale (28). The total score was between 20 and 140, with the higher the score, the higher the level of empathy.

### The Willingness to Fulfill the Contract Scale

The Willingness to Fulfill the Contract Scale was developed by Zhang Xuewen based on the Chinese Medical Personnel Turnover Intention Scale, with an overall Cronbach’s *α* coefficient of 0.699 (6). The scale covers four items: “I often think of leaving the government-funded medical profession and switching to other medical fields (such as non-directed general clinical specialties and basic medicine),” “I often think of giving up studying medicine,” “During the contract compliance period, I will leave the rural designated signing unit and go to a city hospital,” and “During the contract compliance period, I will leave the medical profession.” The scale used a 6-point Likert scale, with a range from 1 (strongly agree) to 6 (strongly disagree), for a total score of 4–24. The higher the score, the stronger the willingness to fulfill the contract.

### Toronto Alexithymia Scale

Alexithymia is a subjective personality pattern that is difficult to measure quantitatively. Although some questionnaires have been used by previous studies to measure alexithymia, they are usually of low reliability and validity and of poor quality. The TAS-20 Alexithymia Scale was developed by Bagby and Taylor of the University of Toronto in 1984 and has good reliability and validity, revised into a Chinese version by Jinyao et al. (29). TAS-20 has a total of 20 items, divided into three dimensions that detect difficulty in emotional perception, difficulty in emotional expression, and difficulty in extroverted thinking. A 5-point Likert scale was used, ranging from 1 (completely disagree) to 5 (completely agree), with a total score of 20 to 100. Cronbach’s *α* coefficient was 0.83. The higher the score, the more severe the affective disorder, with more than 60 classified as hyper-alexithymia and less than 52 classified as normal range (30).

### Statistical analysis

First, exploratory factor analysis (EFA) and the *α* reliability coefficient method were used to evaluate the reliability and validity of the whole questionnaire scientifically and accurately. Second, descriptive statistical methods were used to describe the socio-demographic and sociological characteristics (component ratio), communication skills attitude, empathy, willingness to fulfill contracts, and alexithymia (mean and standard deviation) of 495 RTMSs for descriptive analysis. Third, the Pearson correlation method was used to observe the correlation between the measured values, and the correlation coefficient was quantified. Finally, on the basis of the above research results, the structural equation model (SEM) was constructed to study the interaction and linkage mode among RTMSs’ communication skills, attitude, empathy, contract fulfillment intention, and alexithymia, as well as the mediating role of empathy. The proposed conceptual model is shown in Figure 1. The feasibility of the conceptual model was verified by constructing a guiding robust maximum likelihood estimation model (MLR) and using several key indicators to measure the degree of data fitting. The indexes included the normalized fit index (NFI), goodness of fit index (GFI), comparative fit index (CFI), adjusted goodness of fit index (AGFI), incremental fit index (IFI), and Tucker–Lewis index (TLI), all of which were greater than 0.90, and the approximate root mean square error (RMSEA) was 0.055. A value of less than 0.8 proved that the model is consistent with the data and assumptions and is an acceptable model.

### Reliability and validity

The results of exploratory factor analysis (EFA) showed that the KMO (Kaiser–Meyer–Olkin) value for the entire questionnaire was 0.874, greater than 0.70. The Bartlett’s test of sphericity was significant (*χ*^2^ = 16897.732, *p* < 0.001). In the factor loading analysis, the varimax orthogonal rotation was performed using the maximum variance method, and the results of the factor loading matrix after rotation proved that the eigenvalue of each of the four evaluation indicators was significantly greater than 1, and the validity of the questionnaire was good. The most commonly used reliability measurement method, the *α* reliability coefficient, also known as the internal consistency coefficient, was used to measure the reliability of the scales covered by the research, and the results were as follows: the Cronbach’s *α* values of Communication Skills Attitude Scale (CSAS), Willingness to Fulfill the Contract Scale, Psychometric Properties of the Toronto Alexithymia Scale (TAS-20), and Jefferson Scale of Physician Empathy-Student Version (JSPE-S) were up to 0.695, 0.723, 0.835, and 0.913, respectively, indicating good reliability.

## Result

### General demographic and academic characteristics

The mean age of the RTMSs was 21.18 ± 1.082 years, of which 45.7% were men and 54.3% were women. The proportion of rural students was 56.6%, higher than that of urban students (43.4%). 34.5% of the participants were only children. 21.4% of the respondents achieved the general clinical score of the college entrance examination (non-public funded and non-rural employment), much higher than the admission score for the RTMSs.

Regarding the reasons for choosing the RTME program, 31.1% of the students said it was because they loved medicine, while 38% had no employment pressure. 79.4% were willing to fulfill the contract with primary healthcare institutions after graduation. Regarding the factors that affected their service obligations, 67.9% of the participants believed that salary was the main factor affecting their future work in rural primary healthcare institutions, as shown in Table 2.

**Table 2:** General characteristics of the respondents (*N* = 495).

General characteristics	*N*	%
Sex		
Male	226	45.7
Female	269	54.3
Grade		
Senior	12	2.4
Middle	371	75
Junior	112	22.6
Reasons for applying for RTMSs		
Love medicine	177	35.7
Ease the burden	32	6.5
No employment pressure	188	38.0
Involuntary choice	67	13.5
Optional	31	6.3
Whether the score reached the general clinical professional score line		
Yes	106	21.4
No	389	78.6
Whether the work place is the place of domicile		
Yes	244	49.3
No	251	50.7
Postgraduate entrance examination intention		
Yes	444	89.7
No	8	1.6
Uncertain	43	8.7
Factors affecting performance		
Salary	336	67.9
Work prospect	24	4.8
Work content	71	14.3
Promotion space	52	10.5
Other factors	12	2.4

### Descriptive analysis of the research variables

The total scores of empathy, alexithymia, and willingness to fulfill the contract were 111.54 ± 15.38, 51.66 ± 10.82, and 18.41 ± 4.13. The total scores of positive attitude and negative attitude of communication skills were 51.93 ± 6.85 and 33.34 ± 6.25, respectively. The detailed sub-dimension statistical results of each indicator are shown in Table 3. The assessment scores were divided into low, moderate, and high levels using the interquartile range method. In terms of assessment scores, 136 students (27.47%) possess a low level of PAS, whereas 157 students (31.71%) exhibit a low level of NAS. Meanwhile, 248 students (50.10%) demonstrate a moderate level of PAS, and 237 students (47.88%) show a moderate level of NAS. Furthermore, 111 students (22.42%) possess a high level of PAS, compared to 101 students (20.40%) who exhibit a high level of NAS.

**Table 3:** Item scores in communication skills attitude, empathy, alexithymia, and willingness to fulfill the contract.

Items	Mean ± SD
Jefferson Scale of Physician Empathy-Student Version (JSPE-S)	111.54 ± 15.38
Opinion-taking factor	56.21 ± 8.00
Compassionate care factor	43.66 ± 6.63
Transposition factor	11.67 ± 2.12
Toronto Alexithymia Scale	51.66 ± 10.82
Difficult to recognize your emotions	17.81 ± 5.51
Difficult to describe one’s feelings	13.65 ± 3.94
Externally oriented thinking	20.19 ± 3.94
Willingness to Fulfill the Contract Scale	18.41 ± 4.13
I often want to leave the free medical major and transfer to other medical majors (such as basic clinical)	4.33 ± 1.54
I often want to give up studying medicine	4.63 ± 1.42
During the future performance of the contract, I will leave the directional contracting unit	4.31 ± 1.52
In the future, I will leave the medical industry	5.14 ± 1.07
Communication Skills Attitude Scale	85.28 ± 7.67
Positive attitude subscale	51.93 ± 6.85
Negative attitude subscale	33.34 ± 6.25

Reference ranges: Toronto Alexithymia Scale (TAS-20) = 51.55 ± 10.82 (35); Jefferson Scale of Physician Empathy (JSPE-S) = 116.54 ± 10.85 (38); Communication Skills Attitude Scale: negative attitude subscale (CSAC-NAS) = 33.0 ± 7.3; Communication Skills Attitude Scale: positive attitude subscale (CSAS-PAS) = 51.3 ± 7.3 (11).

### Correlations of study variables

The Pearson correlation coefficients of the four main observed variables in the study are reported in Table 4. Alexithymia was negatively correlated with willingness to fulfill the contract, empathy, and communication skills attitude, while the willingness to fulfill the contract was positively correlated with empathy and communication skills attitude. Empathy was also positively correlated with communication skills attitude.

**Table 4:** Correlation coefficients among study variables.

	Alexithymia	Willingness to fulfill the contract	Empathy
Alexithymia			
Willingness to fulfill the contract	−0.192**		
Empathy	−0.356**	0.262**	
Communication Skills Attitude	−0.091*	0.109*	0.256**

### Testing of the constructed study model

**Figure 2:**
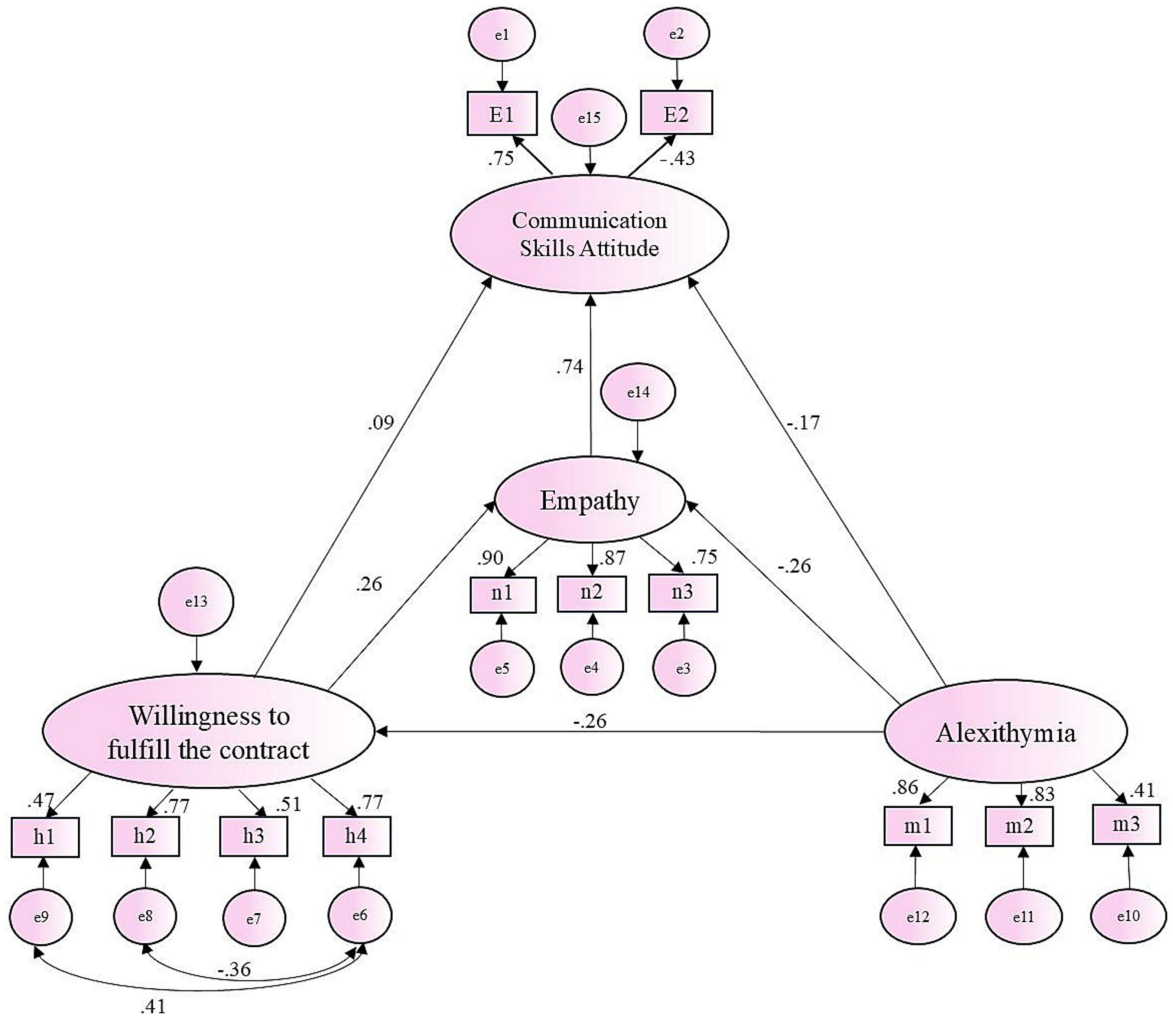
Final model and standardized model path, the mediation analysis path, and the effect value.

The SEM was used to connect, observe, and evaluate the relationships between the four variables (empathy, alexithymia, willingness to fulfill the contract, and communication skills attitude). The data were fitted to the theoretical model using the optimized generalized least squares method based on the precision model, and the model was modified and improved based on the final fitting indicators. Finally, the constructed model showed the mutual path validity relationships between the four variables, as shown in Figure 2. Through confirmatory factor analysis, the fitting index of the final modified hypothetical model was NFI = 0.919, IFI = 0.49, CFI = 0.949, TLI = 0.938, and RMSEA = 0.055. All indicators meet the requirements of reference values, indicating that the model is well-fitted. The maximum likelihood estimation method was used to correct the deviation of each path 2000 times and guide repeatedly.

Figure 2 and Table 5 show the mediation analysis path and effect value. Alexithymia has a significant direct negative impact on empathy (*β* = −0.26, *p* < 0.001) and a negative impact on communication skills attitude (*β* = −0.17, *p* < 0.001). Empathy has a direct positive impact on communication skills attitude (*β* = 0.74, *p* < 0.001). Willingness to fulfill the contract has a significant direct positive impact on empathy (*β* = 0.26, *p* < 0.001), and a positive impact on communication skills attitudes (*β* = 0.09, *p* < 0.001).

**Table 5:** Significance test of the mediating test.

Model pathways	Estimated	95% CI
Total effects		
Communication skills attitude ← alexithymia	−0.43	(−0.59)–(−0.29)
Communication skills attitude ← willingness to fulfill the contract	0.28	0.13–0.44
Communication skills attitude ← empathy	0.74	0.60–0.92
Empathy ← alexithymia	−0.33	(−0.44)–(−0.21)
Empathy ← willingness to fulfill the contract	0.26	0.12–0.39
Willingness to fulfill the contract ← alexithymia	−0.26	(−0.39)–(−0.14)
Direct effects		
Communication skills attitude ← alexithymia	−0.17	(−0.30)–(−0.05)
Communication skills attitude ← willingness to fulfill the contract	0.09	(−0.03)–(−0.22)
Communication skills attitude ← empathy	0.74	0.60–0.92
Empathy ← alexithymia	−0.26	(−0.37)–(−0.15)
Empathy ← willingness to fulfill the contract	0.26	0.12–0.39
Willingness to fulfill the contract ← alexithymia	−0.26	(−0.39)–(−0.14)
Indirect effects		
Communication skills attitude ← alexithymia	−0.26	(−0.39)–(−0.17)
Communication skills attitude ← willingness to fulfill the contract	0.19	0.09–0.32
Empathy ← alexithymia	−0.07	(−0.14)–(−0.03)

Table 6 presents the quantitative results of two mediation paths between four variables. In the mediating path between willingness to fulfill the contract and communication skills attitude, empathy has a significant mediating effect (95% CI: 0.660–1.753). In the mediating path between alexithymia and communication skills attitude, empathy also has a significant mediating effect [95% CI: (−0.900)–(−0.329)].

**Table 6:** Significance test of every mediating pathway.

Model pathways	95% CI
Communication skills attitude ← empathy ← willingness to fulfill the contract	0.660–1.753
Communication skills attitude ← empathy ← alexithymia	(−0.900)–(−0.329)

## Discussion

“Strengthening rural grassroots construction” is vital for China’s healthcare reform. The key to enhancing rural medical services is to train qualified rural general practitioners who can “go, stay, and be effective” (31). Although the RTME policy has partially alleviated the shortage of medical personnel in rural areas, there are still obvious problems in the quality of students being trained and the specific implementation programs. This study showed that 244 (49.3%) RTMSs had a poor attitude toward communication skills, with a negative attitude score of 33.34 ± 6.25 points, which was higher than that of non-RTMSs (33.0 ± 7.3 points) (11). In rural hospitals, communication skills are crucial for establishing a harmonious doctor–patient relationship, improving medical quality and efficiency, optimizing patients’ medical experience, and enhancing rural doctors’ professional identity, sense of belonging, and sense of honor, and are also key measures for implementing and executing RTME policy. The important and unique value of this study is not only that we selected future doctors in rural hospitals with relatively poor resource quality as research subjects, but also that for the first time, four variables—communication skills attitude, empathy, alexithymia, and willingness to fulfill contract—were included in the structural equation model. On this basis, some suggestions are put forward to improve RTME.

The structural equation model shows that RTMSs’ willingness to fulfill the contract not only has a direct positive effect on communication skills attitude, but also has an indirect positive effect through the mediation of empathy. RTMSs’ willingness to fulfill the contract directly reflects their willingness to serve in rural medical institutions after graduation, which is closely related to the vital health interests of Chinese rural residents and affects the sustainable development of rural medical and health services (32).

In this survey, 102 RTMSs (20.6%) are not willing to fulfill their contract to serve in rural areas after graduation, and even if they fulfill their contract, only 50 RTMSs (10.1%) are willing to continue to work in rural areas after their service expires. As a result, the overall score of the propensity to perform contracts has dropped from 19.01 ± 4.36 points in 2022 to 18.41 ± 4.13 points now (6). The backward development of rural medical institutions, the awareness of limited personal development space, and the low social recognition of rural medical services have led to the wavering willingness and determination of many rural doctors to work in rural areas, which is the main reason for their low willingness to fulfill contracts. According to the path shown in the structural equation model, RTMSs’ low willingness to fulfill contracts may affect their motivation to learn communication skills during undergraduate study, which further results in RTMSs being unable to be well qualified for future rural doctor positions, especially unable to empathize with the feelings and needs of rural residents after illness, and unable to provide them with warm care and support. Finally, the doctor–patient relationship is strained, which affects the effectiveness of doctor–patient communication and the quality of medical services.

Characterized by a lack of imagination and attention to the inner world, alexithymia is a maladaptive psychological phenomenon; severe alexithymia can lead to individual cognitive impairment (33). In the process of medical students learning communication skills, emotional expression, and perception ability are indispensable factors that can help individuals better understand the emotional needs of others, so as to more accurately express their own thoughts and feelings. At the same time, emotional expression and perception also play a crucial role in the establishment of empathy, allowing individuals to experience the emotional world of others more deeply, thus establishing a deeper empathic relationship. As future medical workers, the ability of medical students to identify and express emotions, regulate emotions, and manage pressure not only directly affects their own mental health but also affects their ability to empathize with patients, communicate with colleagues and patients. Therefore, due to the heavy academic pressure and strict professional requirements of medical students, the incidence of alexithymia among medical students is higher than that of students in other majors (34). In this study, the average score of RTMSs with alexithymia (51.66 ± 9.12) was higher than that of ordinary medical students (51.55 ± 10.82) (35), which may be related to the high academic intensity, long learning time, high economic pressure, heavy family burden, and limited career development opportunities after their service period. The results of the structural equation model show that alexithymia of RTMSs can affect their communication skills, attitude directly and indirectly through empathy. The future treatment group of RTMSs is mainly villagers with low education levels, and the elderly account for the majority. In this case, the negative attitude of communication skills and lack of empathy caused by high alexithymia will have a negative impact on service efficiency, treatment effect, and doctor–patient communication. For example, communication barriers caused by alexithymia may lead to villagers’ misunderstanding of the condition, thus affecting their compliance with treatment. It will also cause doctors to be unable to truly understand the pain and needs of villagers, reducing the treatment effect and villagers’ medical satisfaction.

Empathy is the ability to experience another person’s situation from their point of view in order to feel and understand their emotions. A large number of studies have shown that empathy is essential for building a trust network in doctor–patient communication and is crucial for efficient clinical diagnosis and treatment (16, 36). According to the results of the structural equation model in this study, empathy can not only directly have a positive impact on communication skills attitude, but also serve as the intermediary between “willingness to fulfill contract” and “communication skills attitude,” and between “alexithymia” and “communication skills attitude.” Empathy affects communication skills from the following aspects: First, in the medical environment, effective listening is the first step for doctors to gain the trust of patients and establish a good doctor–patient relationship. Medical students with strong empathy are more likely to take the initiative to listen, so as to understand patients’ needs and feelings more accurately. Second, empathy enables medical students to express their understanding and care for patients more appropriately and effectively, and to convey treatment information, explain the condition, and give emotional support in a language and way that is easier for patients to understand, reduce communication barriers, and improve patients’ acceptance and cooperation with treatment. Finally, medical students with strong empathy pay more attention to patients’ feedback in communication, and are able to keenly detect patients’ emotional changes and make appropriate responses. These feedback skills help to establish more positive interactions and enhance patients’ trust and satisfaction.

This study found that the empathy score of RTMSs (111.54 ± 15.38) was higher than that of clinical medical students (102.07 ± 15.76) (37), which may be due to the fact that most of the students who chose to become as RTMSs had a strong sense of humanism, professional aspirations, and lofty ideals, and were genuinely committed to the development of the rural primary medical system, or because a large proportion of the students in this major (56.6%) were from rural areas and were well aware of the difficult and expensive situation of seeking medical care in rural areas, and were eager to change the situation in their hometowns. The mean of RTMSs was close to the reference value of the Jefferson Empathy Scale (116.54 ± 10.85) (38) and was also close to the results of many domestic scholars’ investigations into the empathy ability of clinical medicine students (39, 40). However, this score was inferior to that of clinical medical students in other countries such as the United States, India, and Japan (38, 41, 42), which may be because there are defects in the medical student training program in China, including the lack of training in clinical-related experience and the establishment of humanistic care emotions.

In summary, this study revealed two influence paths of RTMSs’ communication skills attitude by using a structural equation model. Empathy, alexithymia, and willingness to fulfill the contract serve as warning factors and can accurately predict the good or bad attitude of RTMSs toward communication skills; it also shows that empathy has a significant mediating effect on willingness to fulfill the contract influencing communication skills attitude, and on alexithymia, influencing communication skills attitude.

### Implications

The research findings show that for RTMSs: (1) empathy has a positive predictive effect on communication skills attitude; (2) willingness to fulfill the contract has a positive predictive effect on communication skills attitude; (3) alexithymia have a negative predictive effect on communication skills attitude; (4) empathy serves as a mediator between willingness to fulfill the contract and communication skills attitude; (5) empathy serves as a mediator between alexithymia and communication skills attitude. It can be seen that helping RTMSs overcome alexithymia and enhance the willingness to perform the contract is not only the key to establishing a good communication skills attitude, but also an effective way to improve the level of rural medical services, strengthen the construction of rural general medicine teams, and improve the medical conditions of villagers. Accordingly, we propose the following countermeasures:

### Strengthen the RTMSs’ education of professional ethics, professional identity, and career identity to overcome alexithymia

In addition to affecting the communication skills and attitudes of medical students, alexithymia can also lead to the decline of cognitive functions such as memory and attention (43), and even cognitive dysfunction of decision-making in severe cases (44). Previous studies of this research group also found that rural doctors with a high level of alexithymia were more likely to have a high level of job burnout (45). In order to promote the sustainable development of RTME, the Medical University can help RTMSs overcome alexithymia in the following ways. First, strengthen the education of professional ethics, clarify the code of professional ethics, enhance the sense of responsibility and mission of RTMSs, and form a positive professional attitude. Second, it can enhance the empathic ability of RTMSs and improve their ability to recognize and express emotions, thus overcoming alexithymia. Third, RTMSs should be encouraged to actively understand, care for, and meet the emotional needs of rural patients by educating them on professional identity and professional identity, thereby reducing the risk of their own alexithymia.

### The school is constantly improving the teaching model and methods to improve the communication skills and attitudes of RTMSs

Improving RTMSs’ communication skills attitude is an important part of medical education as it directly affects their ability to communicate effectively with patients, colleagues, and healthcare teams in the future. Schools and teachers can integrate communication principles, medical ethics, and interpersonal relationships into curriculum design so that students can understand the importance of good communication in medical practice from a theoretical perspective. Through interesting teaching methods such as case analysis and role playing, students can intuitively feel the impact of different communication methods on patient satisfaction, treatment effect, and doctor–patient relationship, thereby arousing students’ enthusiasm for learning and helping students establish a good attitude toward learning communication skills.

### Strive to enhance RTMSs’ empathy skills actively

The professional characteristics of RTME require that RTMSs should have higher humanistic literacy, communication ability with patients, and team service ability, so as to better understand and respond to the future medical needs of rural patients. However, the cultivation of empathy ability of clinical medical students in China mainly focuses on special lectures and theoretical teaching of humanities courses, and lacks clinical empathy ability practice. At the same time, the proportion of humanities courses in medical schools is small, and most of them are elective courses (46), which makes students less involved and active, which is not conducive to the cultivation of empathic ability. In order to solve this practical problem, schools and educators should enrich teaching forms in the process of cultivating empathy skills in RTMSs, set up empathy education courses, and improve teaching quality by combining theory and practice, so as to improve the overall empathy skills of RTMSs (37).

### Strengthen RTEM policy support and constraint measures to improve the willingness to fulfill the contract of RTMSs

Relevant policies should be formulated by the government and medical institutions to support the career development of rural doctors in targeted employment and stabilize the team of rural doctors. First of all, improve the social status, economic benefits, and development space of rural doctors, and ensure that their salaries are commensurate with their professional skills and workload, that is, attract and retain talents through a reasonable salary system. Second, provide comprehensive social security and welfare benefits for rural doctors to increase their sense of belonging and sense of security. Third, rural doctors should be regularly organized to participate in professional skills training and academic exchange activities to improve the professional skills and professional ethics of medical personnel. Finally, improve the promotion mechanism, provide rural doctors with a clear career promotion path and development space, and encourage them to work at the grassroots level for a long time. At the same time, it is necessary to strengthen the supervision and management of the implementation process of the RTME policy, discover and correct the existing problems and deficiencies in time, expose and punish the behaviors that violate the policy provisions, and promote the effective implementation of the RTME policy.

### Strengthen social publicity and public opinion guidance to enhance public awareness and recognition of RTME policies

The favorable public opinion operation environment of the RTME policy needs to be created by TV media, short videos, and other means to guide the public to correctly understand the importance and value of the RTME profession. At the same time, the government needs to strengthen public health education, guide the public to correctly understand the importance of rural medical care and the development gap, and enhance the public’s trust and support for rural general practitioners. Rural primary medical institutions can enhance the social recognition and reputation of rural residents to the medical team through community publicity and public welfare activities, and promote the implementation of the RTME project through a positive social atmosphere.

### Advantages of the research

This study employed a combination of multilevel random sampling and cross-sectional analysis to obtain a representative sample (*N* = 495) from three medical universities in Shandong Province. This approach provided a national cross-sectional snapshot of government-funded RTMSs during a policy-critical period. A sequential analytical pipeline was implemented: one-way ANOVA (for group comparisons), exploratory factor analysis (EFA, for structural validation), bivariate correlation analysis (for path screening), and structural equation modeling (SEM). This methodological sequence offers four key advantages: (1) ensuring robust construct validity through measurement verification prior to modeling; (2) estimating multiple path effects while controlling for measurement error; (3) identifying specific targets for early curricular interventions; (4) evaluating overall model fit. The SEM framework simultaneously revealed direct and indirect effects among variables, generating robust and policy-actionable coefficients unattainable through conventional cross-sectional bivariate analyses. Consequently, this research provides schools and policymakers with a scientific early-warning indicator. It clearly delineates the critical evidence base for cultivating RTMSs who not only fulfill their contractual obligations but also possess the competencies—such as communication skills—essential for sustainable service in rural communities.

### Limitations and future directions

Although this study has a unique contribution and significance for the construction of the rural medical system and the stability of the rural doctor team, there are also some limitations. First of all, the current domestic academic research on RTMSs’ cultivation is insufficient, and there are few comparable data and factors. Second, since this study is a cross-sectional survey and lacks follow-up dynamic tracking, follow-up longitudinal studies are needed to verify our research results. In future, from the perspective of predictive factors, we will accurately construct a comprehensive and practical intervention plan for the key factors that affect the attitude of communication skills, and guide RTMSs to develop in a more positive and efficient direction.

## Data Availability

The original contributions presented in the study are included in the article/Supplementary material, further inquiries can be directed to the corresponding authors.

## References

[1] Department of Finance of the People’s Republic of China. (2024). Policy interpretation of guiding opinions on promoting healthy village construction. Available online at: https://www.gov.cn/zhengce/zhengceku/202409/content_6972154.htm (Accessed September 10, 2024).

[2] Zerun Z Fei L. Study on the current situation and influencing factors of the elderly disability in rural China. Chin Health Serv Manage. (2024) 41:300–6.

[3] Nian Z Changyin Y Jiming Z Fei L Lingli C Haifeng P. Research hotspots and trends on the training of targeted admission medical students in China. Chin Gen Pract. (2023) 26:8. doi: 10.12114/j.issn.1007-9572.2022.0233

[4] Reply to Recommendation No. 3659 of the Second Session of the 14th National People’s Congress. Available online at: https://www.nhc.gov.cn/wjw/jiany/202409/f5565bd54857460293432ad84ff0f7e7.shtml (Accessed September 10, 2024).

[5] Yang W Shaoqun Z Beizhong L Fang G Zhi D. The relevant factors influencing directional medical students serve for community. Chin Gen Pract. (2014) 25:2996–3000. doi: 10.3969/j.issn.1007-9572.2014.25.27

[6] Xuewen Z Bing S Zhuang T Bin Y Chao W Ying Z. Relationship between honesty-credit, specialty identity, career identity, and willingness to fulfill the contract among rural-oriented tuition-waived medical students of China: a cross-sectional study. Front Public Health. (2023) 11:1089625. doi: 10.3389/fpubh.2023.1089625 37529424 PMC10388187

[7] Core Committee, Institute for International Medical Education. Global minimum essential requirements in medical education. Med Teach. (2002) 24:130–5. doi: 10.1080/01421590220120731 12098431

[8] Leijun L Xiaowei X Nianhong G Qinling W. The application of SEGUE frame situational case teaching method in the cultivation of doctor-patient communication ability of medical students. China Contin Med Educ. (2024) 16:82–6.

[9] Xiaoqi C Jie L Jiazhong S Hong L Haohua D. Study on the training of doctor-patient communication skills for medical students based on the foreign experiences. Chin J Med Educ. (2009) 29:51–2. doi: 10.3760/cma.j.issn.1673-677X.2009.01.019

[10] Enran C Ying S Yunning W Siyun W. The role of a ladderlike communication skill course on fostering doctor-patient communication competence of students in rural-oriented free tuition medical education program. Chin Gen Pract. (2024) 27:1561–7. doi: 10.12114/j.issn.1007-9572.2023.0215

[11] Yingzhen S Dongjun Z Junxia R Bo L Huijun L Chunxiao S. Relationship between professional commitment, core self-evaluation and attitude towards communication skills in medical college students. J Xinxiang Med Coll. (2016) 33:881–6. doi: 10.7683/xxyxyxb.2016.10.012

[12] Peishu R Jianping S Xiaoqing Y Xiaojun W Zhilan Y. A study on the reliability and validity of the Chinese version of communication skills attitude scale. Chin J Nurs Educ. (2012) 9:215–7. doi: 10.3761/j.issn.1672-9234.2012.05.007

[13] Feng D Yunwei OY Zhong W Qiang Y. Survey on the doctor-patient communication education of medical student. Chongqing Med. (2015) 44:1241–3. doi: 10.3969/j.issn.1671-8348.2015.09.029

[14] Shiyu H Lei H Wei F Qingwei L Yan F. The correlation between positive attitude towards communication skills and empathy ability among medical students. Chin J Med Educ. (2018). 7. doi: 10.26914/c.cnkihy.2018.030727

[15] Juan W Li L Wenjuan L Feng W Duanying C Hongping Z. Empathy: a new perspective of improving doctor-patient communication. Med Philos. (2011) 32:25–26+29.

[16] Frans D Bensing A Lagro-Janssen J. Effectiveness of empathy in general practice: a systematic review. Br J Gen Pract. (2013) 63:e76–84. doi: 10.3399/bjgp13X660814 23336477 PMC3529296

[17] Junfang G Lihong J Xiuzhen W. Effect of head nurses' empathic ability on nurses' job satisfaction and turnover intention. Int J Nurs. (2018) 37:3. doi: 10.3760/cma.j.issn.1673-4351.2018.23.010

[18] Moriguchi Y Decety J Ohnishi T Maeda M Mori T Nemoto K. Empathy and judging other’s pain: an fMRI study of alexithymia. Cereb Cortex. (2007) 17:2223–34. doi: 10.1093/cercor/bhl130 17150987

[19] Banzhaf C Hoffmann F Kanske P Fan Y Walter H Spengler S. Interacting and dissociable effects of alexithymia and depression on empathy. Psychiatry Res. (2018) 270:631–8. doi: 10.1016/j.psychres.2018.10.045, PMID: 30384283

[20] Hrtwig EA Hrtwig S Aust HR Heekeren I Heuser I. No words for feelings? Not only for my own: diminished emotional empathic ability in alexithymia. Front Behav Neurosci. (2020) 14:112. doi: 10.3389/fnbeh.2020.00112 33061894 PMC7517829

[21] Lyvers M Randhawa A Thorberg FA. Self-compassion in relation to alexithymia, empathy, and negative mood in young adults. Mindfulness. (2020) 11:1655–65. doi: 10.1007/s12671-020-01379-6

[22] Yaru LSW. A national survey on the obstacles to career development of college students in China. Res Social Chin Character. (2007) 5:50–4. doi: 10.3969/j.issn.1006-6470.2007.05.010

[23] Rees C Sheard C Davies S. The development of a scale to measure medical students' attitudes towards communication skills learning: the communication skills attitude scale (CSAS). Med Educ. (2002) 36:141–7. doi: 10.1046/j.1365-2923.2002.01072.x, PMID: 11869441

[24] Milaniak I Wilczek-Rużyczka E Przybyłowski P. Role of empathy and altruism in organ donation decision making among nursing and paramedic students. Transplant Proc. (2018):1928–32. doi: 10.1016/j.transproceed.2018.02.153 30177082

[25] von Harscher H Desmarais N Dollinger R Grossman S Aldana S. The impact of empathy on burnout in medical students: new findings. Psychol Health Med. (2017) 23:295–303. doi: 10.1080/13548506.2017.1374545 28954529

[26] Hojat M Gonnella JS. What matters more about the interpersonal reactivity index and the Jefferson scale of empathy? Their underlying constructs or their relationships with pertinent measures of clinical competence and patient outcomes? Acad Med. (2017) 92:743–5. doi: 10.1097/ACM.0000000000001424 28557931

[27] Hojat M Gonnella JS Nasca TJ Mangione S Veloksi JJ Magee M. The Jefferson scale of physician empathy: further psychometric data and differences by gender and specialty at item level. Acad Med. (2002) 77:S58–60. doi: 10.1097/00001888-200210001-00019, PMID: 12377706

[28] Tian J Xiaoyan W Yuanyuan L Xiaosong L. Reliability and validity of the Jefferson scale of physician empathy in Chinese medical students. J Sichuan Univ. (2015) 46:602–5. doi: 10.13464/j.scuxbyxb.2015.04.021 26480667

[29] Jinyao Y Shuqiao Y Xiongzhao Z. The Chinese version of the TAS-20: reliability and validity. Chin Ment Health J. (2003) 11:763–7. doi: 10.3321/j.issn:1000-6729.2003.11.011

[30] Seo SS Chung U-S Rim HD Jeong SH. Reliability and validity of the 20-item Toronto alexithymia scale in Korean adolescents. Psychiatry Investig. (2009) 6:173–9. doi: 10.4306/pi.2009.6.3.173, PMID: 20046392 PMC2796065

[31] Jing Z Lan W Qian Y. Investigation on the current situation of general practitioners in a rural primary medical institution. J MuDanJiang Med Univ. (2018) 39:113–5. doi: 10.13799/j.cnki.mdjyxyxb.2018.05.040

[32] Shumeng Y Xuewen Z Chengming Y Bing S Lei S. Professional identity and contract performance tendency of rural-oriented public-funded medical students and their correlation. Chin Rural Health Serv Admin. (2023) 43:356–63. doi: 10.19955/j.cnki.1005-5916.2023.05.011

[33] Huajie S Manli J Chenglin Z Feng W. Review of alexithymia research. Sci Technol Vision. (2016) 27:158–9. doi: 10.19694/j.cnki.issn2095-2457.2016.27.115

[34] Yanlan L Lijuan L Jun Y. The impact of affective disorders on risky behaviors among college students. Chin J Behav Med Brain Sci. (2021) 30:6. doi: 10.3760/cma.j.cn371468-20210624-00344

[35] Zhu Y Luo T Jie L Bo Q. Influencing factors of alexithymia in Chinese medical students: a cross-sectional study. BMC Med Educ. (2017) 17:66. doi: 10.1186/s12909-017-0901-8 28372565 PMC5379661

[36] Neumann M Scheffer C Tauschel D Lutz G Edelhuser F. Physician empathy: definition, outcome-relevance and its measurement in patient care and medical education. GMS Z Med Ausbild. (2012) 29:Doc11. doi: 10.3205/zma000781 22403596 PMC3296095

[37] Tianyi B Wendi Z Guanying N Xiaohui Q Zhengxue Q Jiawei Z. Analysis on status and influencing factors of empathy ability among clinical medical students. Health Vocation Educ. (2024) 42:109–15.

[38] Hojat M DeSantis J Shannon SC Mortensen LH Speicher MR Bragan L. The Jefferson scale of empathy: a nationwide study of measurement properties, under-lying components, latent variable structure, and national norms in medical students. Adv Health Sci Educ Theory Pract. (2018) 23:899–920. doi: 10.20037/j.issn.1671-1246.2024.15.31 29968006 PMC6245107

[39] Yajing Z Jing S Guoqing H Yang H Jiangxiong P Aiguo D. Analysis of empathy ability of medical undergraduates of two medical universities in Hunan province. Anhui Med Pharm J. (2019) 23:2535–8.

[40] Ningxi Y Xiaoyang L Hong Y Shiyue L Qingshan G. Influence of narrative medical education on empathy ability and academic achievement of clinical medical students: a randomized controlled trial. Chin J Clin Psychol. (2018) 26:556–60. doi: 10.3969/j.issn.1009-6469.2019.12.050

[41] Chatterjee A Ravikumar R Singh S Chauhan PS Goel M. Clinical empathy in medical students in India measured using the Jefferson scale of empathy-student version journal of educational evaluation for health professions. J Educ Eval Health Prof. (2017) 14:33. doi: 10.3352/jeehp.2017.14.33, PMID: 29278905 PMC5801322

[42] Son D Shimizu I Ishikawa H. Communication skills training and the conceptual structure of empathy among medical students. Perspect Med Educ. (2018) 7:264–71., PMID: 29671134 10.1007/s40037-018-0431-z PMC6086812

[43] Mingming H. On the relationship between alexithymia and cognitive failure in college students: a serial mediation model. J Leshan Norm Univ. (2024) 39:124–33. doi: 10.1007/s40037-018-0431-z

[44] Zhang L Wang K. Study of decision-making in alexithymia. ACTA Univ Med Anhui. (2013) 48:3. doi: 10.16069/j.cnki.51-1610/g4.2024.04.017

[45] Xuewen Z Xuejia B Min W. The influence of personality, alexithymia and work engagement on burnout among village doctors in China: a cross-sectional study. BMC Public Health. (2021) 21:1507. doi: 10.1186/s12889-021-11544-8 34348678 PMC8335472

[46] Yifen Z Fang S Xian Y Hua X. Analysis on the present situation of medical humanities curriculum in medical colleges and universities in Jiangsu Province. Med J Commun. (2018) 32:3.

