# Supplementary material for: The relationship between alexithymia, empathy, willingness to fulfill the contract, and communication skills attitudes of rural-oriented tuition-waived medical students of China: a cross-sectional study
Source: Front Med (Lausanne). 2025 Sep 26;12:1648789. doi: 10.3389/fmed.2025.1648789 (PMC12511122; doi:10.3389/fmed.2025.1648789)
Supplement: Supplementary file 1 [file Table_1.docx]

**Questionnaire on the relationship between alexithymia, empathy, willingness to fulfill the contract and** **communication skills attitude of rural-oriented tuition-waived medical students of China: a cross-sectional study.**

Dear fellow students:

Hello! In order to understand the current situation of alexithymia, empathy, willingness to fulfill the contract, and communication skills attitude among rural-oriented tuition-waived medical students (RTMSs), and to better cultivate excellent rural medical and health talents to help the development of rural medical and health care, we sincerely invite you to participate in this survey. The credibility of your research results depends on your serious and objective answers to the questions. This survey is anonymous, there are no right or wrong answers. This survey is only for research purposes of this project. We will strictly keep the survey information confidential, please do not have any concerns. Please fill out this questionnaire and read the questions carefully. Your true opinion is very important to us, and we hope to receive your support and assistance. Thank you! Please select your own situation and answer options after each question. (Please **√** under the serial number that matches your situation)

**Table 1 demographic characteristics survey and academic characteristics survey**

| 1 | Age: | 2 | Sex: (1) Male (2) Female |
| --- | --- | --- | --- |
| 3 | Origin of student：(1) Rural area (2) Urban area | 4 | Grade:(1) Junior (2) Middle (3) Senior |
| 5 | Only child of parents: (1) Yes (2) No | 6 | Reached the clinical medical college entrance examination score line: (1) Yes (2) No |
| 7 | Reasons for applying for RTMSs (single option): (1) Love medicine (2) Love primary health care (3) Reduce the burden on the family (4) Future employment is guaranteed (5) Involuntary choice (6) Optional | | |
| 8 | Your designated workplace is your place of domicile: (1) Yes (2) No | 9 | You are currently a member of a social organization/student organization: (1) Yes (2) No |
| 10 | You are currently the class Committee/Youth League Committee: (1) Yes (2) No | 11 | Your current learning goals are clear: (1) Yes (2) No |
| 12 | You are willing to fulfill the contract to work in the grass-roots hospital: (1) Yes (2) No | | |
| 13 | Your Continuing Education program is (single option): (1) After undergraduate graduation, participate in the national unified entrance examination for postgraduate students (2) During the performance of the contract, participate in the part-time graduate admission examination (3) After completing the service, you shall participate in the national unified entrance examination for postgraduate students (4) No intention to take the postgraduate entrance exam (5) You don't know. Just take it one step at a time | | |
| 14 | After the end of your service, your work plan is (single choice): (1) Continue to work in primary health care institutions (2) To the second and above unit development (3) In a non-medical profession (4) You don't know. Just take it one step at a time | | |
| 15 | Factors influencing future work willingness (single choice): (1) Salary and treatment (2) Promotion space (3) Work content and atmosphere (4) Working environment condition (5) Other factors | | |
| 16 | You think being a grassroots rural doctor can achieve life value (professional identity): (1) Yes (2) No | | |

**Table 2 Communication Skills Attitude Scale (CSAS)**

(Please √ on the option that best suits your situation, depending on your actual feelings)

| **Project** | **Completely disagree** | **Disagree** | **Neutral** | **Agree** | **Completely agree** |
| --- | --- | --- | --- | --- | --- |
| 1.In order to be a good doctor, I must have excellent communication skills. |  |  |  |  |  |
| 2.I don't think it's important to learn communication skills |  |  |  |  |  |
| 3.No one can get his medical degree because of lack of communication skills |  |  |  |  |  |
| 4.I think developing communication skills is as important as learning medical knowledge |  |  |  |  |  |
| 5.Learning communication skills helps me respect patients |  |  |  |  |  |
| 6.I don't have time to learn communication skills |  |  |  |  |  |
| 7.Learning communication skills is fun |  |  |  |  |  |
| 8.I don't want to spend time discussing communication skills |  |  |  |  |  |
| 9.Learning communication skills helps to strengthen my teamwork ability |  |  |  |  |  |
| 10.Learning communication skills has improved my ability to communicate with patients |  |  |  |  |  |
| 11.I think the teaching of communication skills complicates the obvious |  |  |  |  |  |
| 12.Learning communication skills is fun |  |  |  |  |  |
| 13.Learning communication skills is too easy |  |  |  |  |  |
| 14.Learning communication skills helps or will help me respect my colleagues |  |  |  |  |  |
| 15.I can hardly believe the knowledge about communication skills introduced by non-professional teachers |  |  |  |  |  |
| 16.Learning communication skills helps me understand the patient's right to privacy and informed consent |  |  |  |  |  |
| 17.If communication skills sound more like scientific topics, they will be more convincing |  |  |  |  |  |
| 18.For those who apply for a medical degree, I think it is a good idea to learn communication skills |  |  |  |  |  |
| 19.I don't have to learn good communication skills to be a good doctor |  |  |  |  |  |
| 20.I find it hard to admit that my communication skills are faulty |  |  |  |  |  |
| 21.I think it's really useful for people who take a medical degree to learn communication skills |  |  |  |  |  |
| 22.What I need to graduate successfully is exam taking ability, not communication ability |  |  |  |  |  |
| 23.Learning communication skills is suitable for medical students |  |  |  |  |  |
| 24.I find it hard to learn communication skills with a serious attitude |  |  |  |  |  |
| 25.Learning communication skills is important because communication skills are a lifetime skill |  |  |  |  |  |
| 26.Learning communication skills should be a matter for psychological students, not for medical students |  |  |  |  |  |

**Table 3 Psychometric properties of the Toronto Alexithymia Scale(TAS-20)**

**(Please √ on the option that best suits your situation, depending on your actual feelings)**

| **Project** | **Completely disagree** | **Disagree** | **Neutral** | **Agree** | **Completely agree** |
| --- | --- | --- | --- | --- | --- |
| 1．I often don't know what I feel inside |  |  |  |  |  |
| 2．I find it difficult to describe my inner feelings in proper words |  |  |  |  |  |
| 3.I have some feelings that even doctors can't understand |  |  |  |  |  |
| 4.I can easily describe my inner feelings |  |  |  |  |  |
| 5.I prefer to analyze problems rather than just describe them |  |  |  |  |  |
| 6.When I feel sad, I don't know whether it's sadness, fear or anger |  |  |  |  |  |
| 7.I often feel confused about some feelings of my body |  |  |  |  |  |
| 8.I often only pay attention to what happened and ignore the reason why it happened |  |  |  |  |  |
| 9.I have some inner feelings that I can't recognize |  |  |  |  |  |
| 10.It's important for me to know my emotional experience |  |  |  |  |  |
| 11.I can't describe how I feel about others |  |  |  |  |  |
| 12.They say I seldom talk about my inner feelings |  |  |  |  |  |
| 13.I don't know what's going on inside me |  |  |  |  |  |
| 14.I often don't know why I'm angry |  |  |  |  |  |
| 15.I prefer to talk with others about their daily activities rather than their inner feelings |  |  |  |  |  |
| 16.I would rather watch a light hearted entertainment film than a serious melodrama |  |  |  |  |  |
| 17.Even close friends, I can't express my deep feelings |  |  |  |  |  |
| 18.I can feel intimacy with someone, even when we are silent |  |  |  |  |  |
| 19.I think it is useful to reflect on my inner feelings to solve personal problems |  |  |  |  |  |
| 20.Looking for hidden meanings in movies or plays can distract people from entertainment |  |  |  |  |  |

**Table 4 Willingness To Fulfill The Contract Scale**

**(Please √ on the option that best suits your situation, depending on your actual feelings)**

| **Project** | **Strongly disagree** | **Comparative disagree** | **A little disagree** | **A little agree** | **Comparative agree** | **Strongly agree** |
| --- | --- | --- | --- | --- | --- | --- |
| 1.I want to leave the specialty of rural-oriented tuition-waived medicine and switch to other medical specialties |  |  |  |  |  |  |
| 2.I often want to give up studying medical specialty |  |  |  |  |  |  |
| 3.In the future, I will leave the orientated rural hospital during the term of my contract |  |  |  |  |  |  |
| 4.In the future, I will leave the medical industry during the term of my contract |  |  |  |  |  |  |

**Table 5 Jefferson Scale of Physician Empathy-student version (JSPE-S)**

**(Please √ on the option that best suits your situation, depending on your actual feelings)**

| **Project** | **Completely disagree** | **Disagree** | **A little disagree** | **Uncertain** | **A little agree** | **Agree** | **Completely agree** |
| --- | --- | --- | --- | --- | --- | --- | --- |
| 1.The doctor's understanding of the patient's and their family's emotions is not helpful to the therapeutic effect (medicine/surgery) |  |  |  |  |  |  |  |
| 2.Doctors' empathy with patients can make patients feel better |  |  |  |  |  |  |  |
| 3.It is difficult for doctors to think from the perspective of patients |  |  |  |  |  |  |  |
| 4.In the relationship between doctors and patients, it is equally important to understand the patient's body language and oral communication |  |  |  |  |  |  |  |
| 5.Doctors' sense of humor helps patients get better clinical treatment effect |  |  |  |  |  |  |  |
| 6.Because everyone has different thoughts and values, it is difficult to look at problems from the perspective of patients |  |  |  |  |  |  |  |
| 7.In the process of medical history inquiry, it is not important to pay attention to the patient's emotional changes |  |  |  |  |  |  |  |
| 8.Paying attention to the patient's personal experience has nothing to do with the treatment outcome |  |  |  |  |  |  |  |
| 9.When providing medical services, we should try to consider problems from the standpoint of patients |  |  |  |  |  |  |  |
| 10.The doctor sympathizes with the patient, and the patient will feel that the treatment is effective |  |  |  |  |  |  |  |
| 11.The disease can only be treated with medicine or surgery, so establishing feelings with the patient has no clear benefits for treatment |  |  |  |  |  |  |  |
| 12.Asking the patient what happened in daily life has no effect on understanding the main complaint of physical symptoms |  |  |  |  |  |  |  |
| 13.The doctor should pay attention to the body language or nonverbal clues revealed by the patient in order to understand the patient's inner thoughts and changes |  |  |  |  |  |  |  |
| 14.I don't think emotion plays a role in the treatment of diseases |  |  |  |  |  |  |  |
| 15.Empathy is a therapeutic skill. Without empathy, it will be difficult to become a successful doctor |  |  |  |  |  |  |  |
| 16.In the doctor-patient relationship, understanding the emotional state of patients and their families is a very important factor |  |  |  |  |  |  |  |
| 17.In order to provide better medical services, doctors should try to consider problems from the perspective of patients |  |  |  |  |  |  |  |
| 18.Doctors should not allow themselves to be moved by the strong emotional relationship between patients and their families |  |  |  |  |  |  |  |
| 19.I don't like reading literature or art books that have nothing to do with medicine |  |  |  |  |  |  |  |
| 20.I believe empathy is an important factor in the treatment process |  |  |  |  |  |  |  |

Investigator： Time of investigation：
